# Primary Staged Bilateral Total Hip Arthroplasty in a Patient With Short Stature and Hartofilakidis Type I Developmental Dysplasia of the Hip

**DOI:** 10.7759/cureus.52710

**Published:** 2024-01-22

**Authors:** Nikolaos Milonakis, Georgios Douvlis, Christothea –Alexandra Tsiridis, Zakareya Gamie, Eustathios Kenanidis, Eleftherios Tsiridis

**Affiliations:** 1 Orthopaedic Department, Papageorgiou General Hospital, Aristotle University of Thessaloniki, Thessaloniki, GRC; 2 Tsiridis Orthopaedic Institute, ICAROS Clinic, Thessaloniki, GRC; 3 Centre of Orthopaedic and Regenerative Medicine (CORE) Center for Interdisciplinary Research and Innovation (CIRI), Aristotle University of Thessaloniki, Thessaloniki, GRC; 4 Institute of Medical and Biomedical Education, St. George’s University of London, London, GBR

**Keywords:** osteochondrodysplasia, hybrid total hip arthroplasty, osteotomy, bilateral total hip arthroplasty, developmental dysplasia of the hip

## Abstract

Syndromes associated with osteochondrodysplasia, short stature, and DDH are rarely reported in the literature. Total hip arthroplasty (THA) in such cases is a complex procedure with a high rate of complications and difficulties. In this case report, we describe the staged bilateral complex primary THA of a patient with the rare occurrence of a syndrome involving osteochondrodysplasia and DDH, highlighting the surgical challenges and importance of the right prosthesis selection.

## Introduction

Developmental dysplasia of the hip (DDH) is one of the most common developmental skeletal diseases [1]. The DDH prevalence displays a broad geographical variability, ranging from 0.1 to 6.6 per 1,000 live births [2]. This complex syndrome encompasses a broad spectrum of growing hip anatomical abnormalities, sharing the femoral head and acetabulum's abnormal relationship, leading to femoroacetabular incongruency and sometimes subluxation and/or dislocation. DDH is one of the primary risk factors for premature hip osteoarthritis, accounting for up to 26% of total hip arthroplasties (THA) in patients aged 40 or younger [3].

Syndromes associated with osteochondrodysplasia, short stature, and DDH are rarely reported in the literature [4]. Total hip arthroplasty (THA) in such patients is a complex procedure with a high rate of complications and difficulties [4]. Distorted acetabular and proximal hip anatomy, previous procedures, leg length discrepancy (LLD), and the patient's young age are common problems. We aim to present the technical considerations of performing a complex primary staged bilateral THA in a rare case of a Caucasian woman who suffered from a syndrome associated with combined short stature and Hartofilakidis type I DDH post bilateral varus osteotomies during her childhood.

## Case presentation

An ambulatory 44-year-old caucasian woman with bilateral DDH was referred to our department due to severe bilateral hip pain aggravated by activity and progressive walking disability. The patient had undergone bilateral varus proximal femoral osteotomies during her childhood to relocate femoral heads in the acetabulum. No further comorbidities were reported. The patient presented with short stature (height 148 cm, weight 55 kg) and a BMI of 25 kg/m2.

On physical examination, the patient had a painful and limited bilateral passive hip range of motion. There was a leg length discrepancy of 4 cm, with the left lower limb being shorter than the right. The patient also had a bilateral Trendelenburg gait pattern and a positive Thomas test bilaterally demonstrating hip flexion deformities of 30 degrees. The Harris Hip Score was 50.

**Figure 1:**
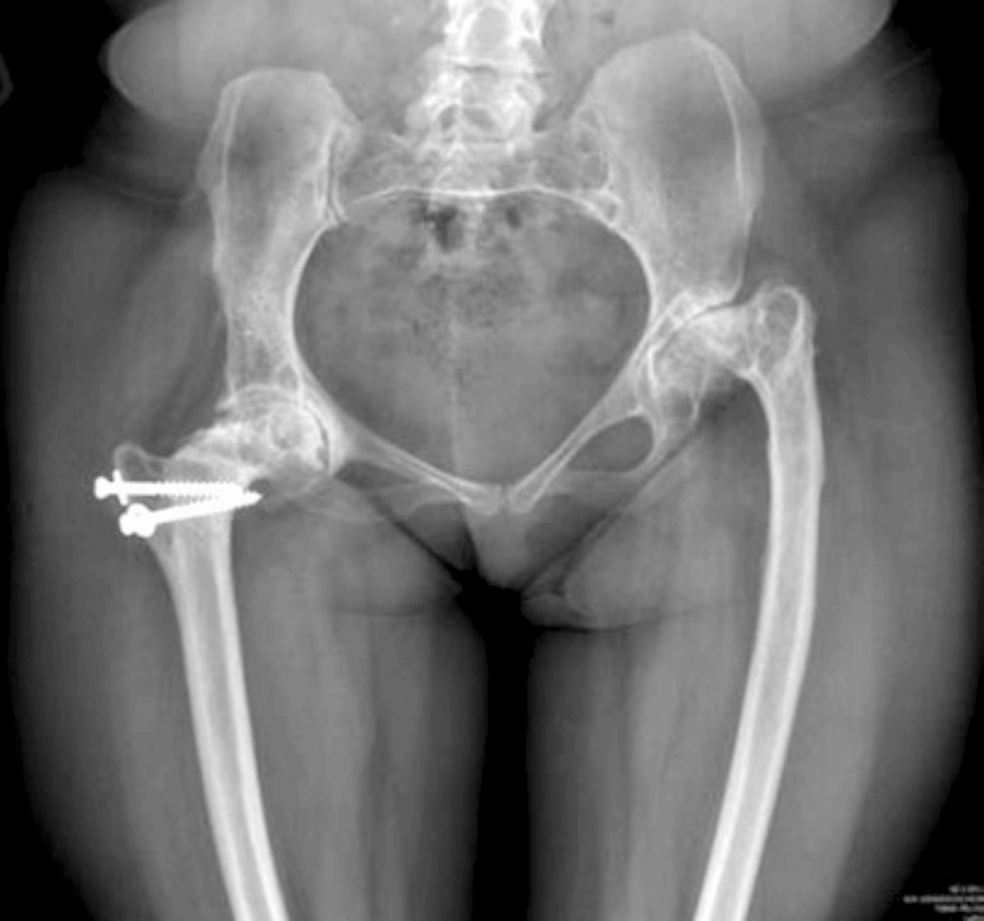
Preoperative anteroposterior view of the pelvis

The radiographic assessment revealed severe osteoarthritic changes with proximal femoral deformity and acetabular dysplasia in both hips. Both acetabulae were shallow and dysplastic (Hartofylakidis type I DDH). The iliac wings were short and square. Coxa vara deformity was present bilaterally post-primary bilateral varus osteotomies at childhood. The right proximal femur contained two partially threaded screws from previous osteotomies; the femoral offset was short and the femoral canal was narrow. The left hip femoral offset was shorter. The femoral canal was also narrow with significant proximal femoral deformity, and a corrective valgus osteotomy was decided preoperatively (Figure 1).

After discussion with the patient about the treatment options, a staged bilateral plan of procedures was selected. Following meticulous radiographic templating, we decided to use cemented Exeter stems to accommodate the narrow femoral canals and the patient's extremely short offset (Stryker Rutherford, New Jersey, Michigan, USA). On both acetabular sides, cemented and uncemented shell options and liner types, including constrained liners, were available.

THAs were performed with the patient in a lateral decubitus position using the posterior approach. Due to the pre-existing scar tissue and subcuticular adhesions from previous osteotomies on both sides and small bilateral offset, special attention was paid to protect the sciatic nerve. Marked wasting of the abductors' mass, with extensive fatty degeneration, was identified bilaterally. Following the hip dislocation, the femoral neck osteotomy was accomplished, and the femur was displaced anteriorly to reveal the acetabulum.

First on the right side, reaming up to a diameter of 45 mm was carefully performed and an uncemented HA-coated acetabular shell, Trident PSL 46 mm (Stryker Corporation, Michigan, USA) was inserted in a neutral position and secured with two screws (6.5 x 20 mm). Attention was then turned to the femoral side. Following the removal of screws, the sclerotic bone around the osteotomy with proximal canal occlusion made reaming almost impossible. A speed diamond-tipped burr was used to penetrate the occlusion and the canal was widened enough to accommodate the 30mm offset broach. Following a third-generation cementing technique, a cemented Exeter V40 femoral stem with a 30mm offset (Stryker Corporation, Michigan, USA) was inserted. A constrained acetabular insert, Trident size D (Stryker Corporation, Michigan, USA) was then inserted into the shell to reduce dislocation risk with the existing abductor insufficiency, and the hip was uneventfully reduced. The hip was stable in all planes, achieving a full range of motion (ROM) and restoration of leg length. The greater trochanter was evaluated intraoperatively and was assessed to be thin; therefore, it was prophylactically reinforced with tension-band wiring in a figure-of-eight manner.

Three months later, the THA on the contralateral left side was undertaken and due to the small acetabular dimension, a metal shell could not be used to achieve press-fit insertion. Therefore, the smallest cemented constrained liner implant available (Trident size D, Stryker Corporation, Michigan, USA) was inserted using the third-generation cementing technique. On the femoral side, lateral bowing of the canal and occlusion by sclerotic bone at the osteotomy site were noted. Based on the preoperative planning, a subtrochanteric straightening, shortening, and valgus osteotomy were performed. The osteotomy was stabilized with a five-holed 3.5 mm small fragment plate and screws penetrating the posterior femoral cortex leaving the canal free for the stem insertion. The proximal femur was reinforced with a femoral strut from the removed segment and cerclage wires to improve stability and healing, and the reaming was efficiently completed. A cemented Exeter 33 mm offset stem (Stryker Corporation, Michigan, USA) was then implanted using the third-generation cementing technique. The hip was uneventfully reduced using a 22 mm head, achieving full and stable ROM with a leg length comparable to the contralateral side.

**Figure 2:**
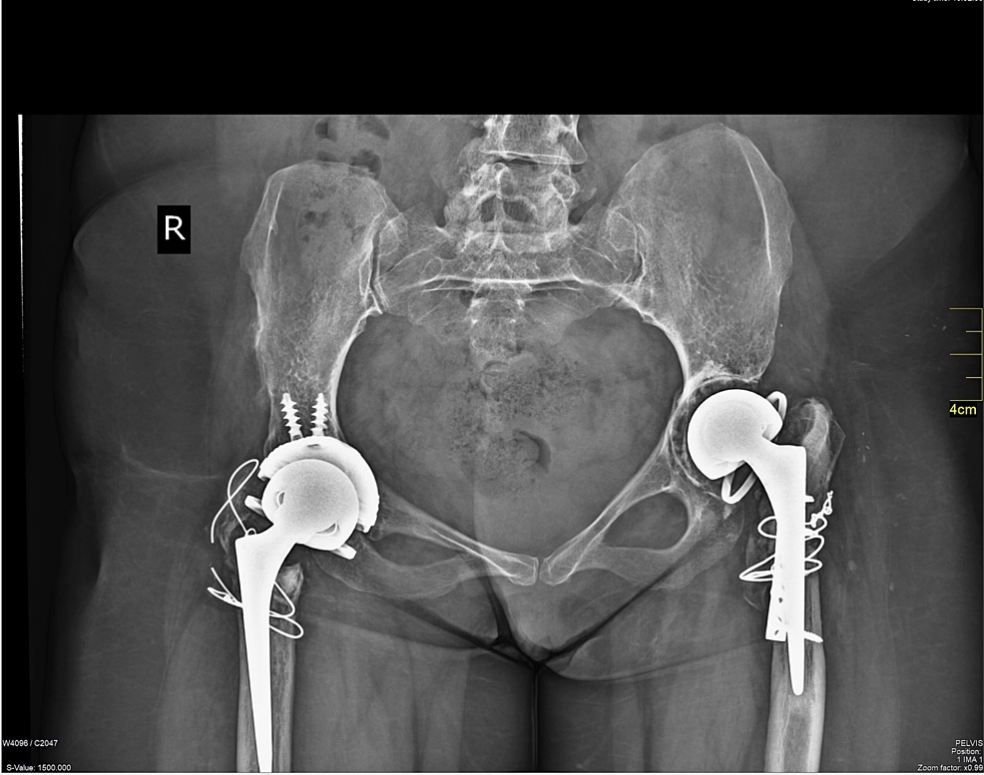
Anteroposterior view of the pelvis 3 years postoperatively

**Figure 3:**
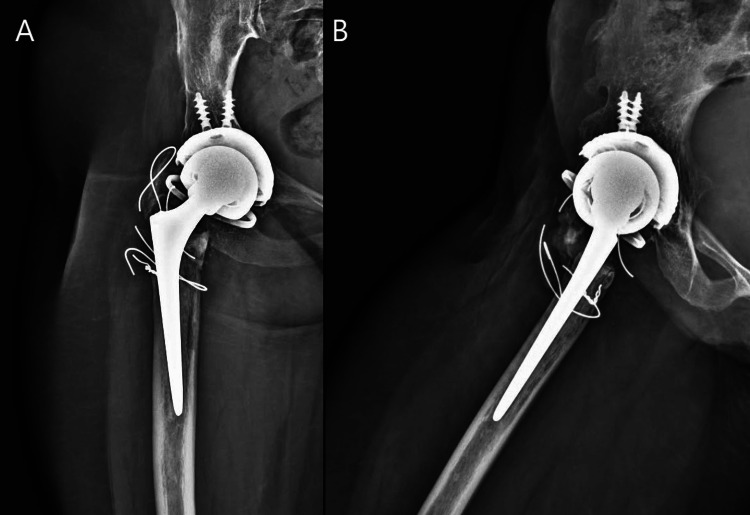
Anteroposterior (A) and lateral (B) views of right hip 4 years postoperatively Uncemented HA-coated acetabular shell (A, B) and cemented Exeter femoral stem (A, B).

**Figure 4:**
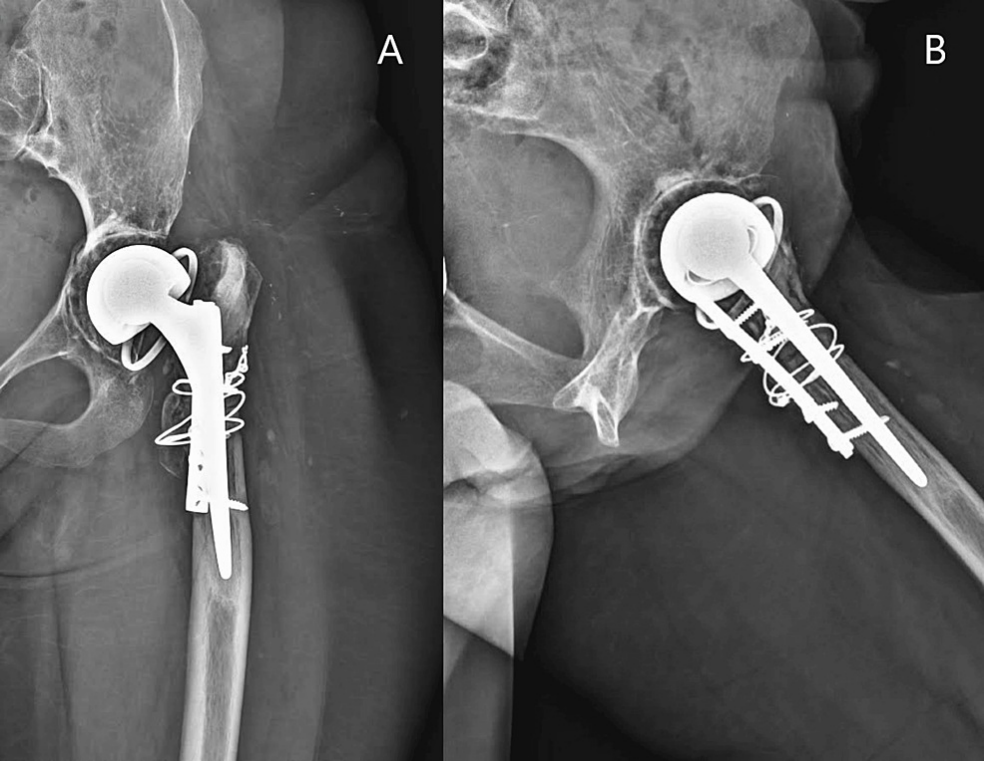
Anteroposterior (A) and lateral (B) views of left hip 4 years postoperatively. Cemented constrained liner (A, B) and cemented femoral Exeter stem (A, B).

Routine antibiotic prophylaxis (teicoplanin 400 mg and cefuroxime 750 mg, one dose preoperatively and 2 doses postoperatively), and thromoboprophylaxis (enoxaparin 4000 IU once per day for 30 days) were administered. The patient was mobilized with partial weight-bearing for eight weeks for each procedure and then as tolerated. There were no adverse events in the early and short-term postoperative period. At the 4-year follow-up, the patient had painless hips bilaterally and a reasonable ROM on both sides. The Harris Hip Score was 94.8, HOOS was 92.9, and WOMAC was 96.1. She mobilized without pain and precautions. The X-rays at years 3 and 4 revealed asymptomatic osteolysis at both, acetabula and femora (Figure 2,3,4).

## Discussion

In this case report, we describe the staged bilateral complex primary THA performed in a patient with the rare occurrence of combined short stature, osteochondrodysplasia, and DDH, highlighting the surgical challenges and importance of the right prosthesis selection.

Conditions with disproportionate short stature can be characterized by altered hip biomechanics and anatomical aberrations, namely the small-sized acetabular and proximal femur size, increasing the complexity of the hip procedure, and highlighting the need for detailed individualized preoperative planning. The disease's facial and vertebral abnormalities may also impede the anaesthesiology plan. Cemented and uncemented stem options, including modular, non-modular, or custom-made stems, have been used for THA in patients with short stature [5]. Good clinical and radiological outcomes and low complication rates have been reported in small groups of patients with short stature undergoing THA with standard cementless or custom femoral implants [5]. Longer operative time and hospitalization, but similar rates of intraoperative fractures, minor complications, and 90-day readmissions and revision compared to matched controls have been reported [6]. However, the proximal femoral deformity in patients with short stature and patients with osteochondrodysplasia has been linked to an increased risk of periprosthetic fractures, femoral perforations, and mechanical failure, especially using uncemented implants [4].

On the other hand, DDH is the most common developmental hip disorder, involving a broad spectrum of femoral and acetabular dysplasia [1]. Typical acetabular deformities include a shallow acetabulum with segmental deficiency of the superior or anterior wall, with the limited bone stock usually rearranged posteriorly [7]. In Hartofilakidis type A acetabulum, as in our case, the acetabulum resembles a normal one. It is generally shallower with segmental deficiency, usually needing a small acetabular component ranging between 38 and 50 mm [8]. Restoration of the hip center of rotation back to the true acetabulum is biomechanically superior, although not always feasible [9,10]. The acetabular augmentation with cement or bone grafting or modular porous metal augments, medialisation of the component with or without medial wall osteotomy, or reinforcement ring are also valuable treatment options with good results [11]. The muscle envelope is impaired with short and malfunctioning abductors, flexors, and extensors, and usually, a marked abundance of capsular ligamentous soft tissue is noticed. Proximal femoral deformities such as the small femoral head, short and narrow femoral neck often with excessive anteversion, small and posteriorly displaced greater trochanter, metaphyseal-diaphyseal mismatch, and increased neck-shaft angle (thus lower offset) are all common DDH characteristics.

The frequently narrow femoral canal, combined with the increased anterior bowing of the proximal femur, may hamper femoral preparation [8,11]. Various stem types, including conical designs with flutes, modular, or custom ones, have been used to address the challenging femoral preparation [12]. The use of a derotational subtrochanteric osteotomy is an alternative solution in the setting of severe anteversion, femoral shortening in cases of high dislocation (Hartofilakidis type C DDH) or for restoration of abductor muscle function, with a reported survivorship of 75% at fourteen years [13]. In any case, avoiding sciatic nerve palsy is of paramount importance; thus, leg lengthening of more than 4 cm is not recommended [14]. Recent reconstruction in unilateral Crowe type 4 deformity, treated with S-ROM stem in combination with subtrochanteric osteotomy, improved leg length with good clinical outcome, and the Harris score reached 77 points at 6 months [15]. 

The bearing surfaces selected in young DDH patients should be designed to reduce wear and improve the THA's longevity [16]. Ceramic on highly cross-linked polyethylene (CoP) and ceramic-on-ceramic (CoC) are usually recommended to reduce the risk of aseptic loosening of the prosthesis [17]. However, ceramic heads are commercially available only after size No. 46-48, limiting surgeons' choices in these cases.

THA for the dysplastic hip has been proven to result in excellent outcomes. Nevertheless, the variety of the aforementioned morphological aberrations, in combination with this population's younger age, inevitably leads to lower implant survival, reported as low as 70% at ten years and 55% at 20 years [18]. Aseptic loosening, postoperative dislocations, polyethylene wear, intraoperative femoral fractures, nerve palsy, distal femoral perforation, and nonunion of the femoral osteotomy have been recognized as the most common complications [8]. In a review of 150 cases of THA in achondroplasia, with varying prostheses and surgical approaches, in comparison to an unaffected matched cohort, a univariate analysis revealed a significant increase in surgical site infection and readmission (3.49 and 2.53 odds ratio respectively in the multivariate analysis) in the immediate postoperative period. However, 95% remained unrevised at 5 years [19]. 

In our case, the primary surgical concerns regarding the acetabular components' implantation were the limited bone stock as well as the small dimensions of the bony structures. Press-fit implantation was only feasible on the left side. Hip anatomy and bone quality did not allow for uncemented shell implantation on the right side. Both uncemented and cemented cup options and small-diameter implants must be available preoperatively in all such cases. Chronic abductor deficiency and small diameter femoral heads that are usually implanted are further issues that had to be considered to reduce postoperative hip instability. Dual mobility and constrained liners must be available in all relevant cases, and if the socket size allows, a dual mobility liner should be used. Previous evidence from our unit has demonstrated that the constrained liner reduces dislocation risk [20]. We used constrained liners in our patient's hips due to the distorted anatomy and extreme deficiency of hip abductors. Constrained liners were implanted in the neutral position to reduce the risk of aseptic loosening. 

On the femoral side, the use of small-diameter cemented Exeter stems gave us the ability to deal with the femoral canal's small dimensions and short hip offset. Furthermore, cemented stems allowed the correction of the excessive femoral neck anteversion, especially on the right side where no osteotomy was performed and fill in the irregular femoral canal, achieving adequate femoral fixation. No other stem design would be capable of restoring such a small offset, except for custom-made implants. The previous varus osteotomies have raised the degree of complexity of the THA on both sides. During the right THA, removing the pre-existing screws and preparing the femoral canal proved challenging even for our choice of the small stem. The thinning of the greater trochanter also created significant concerns, subsequently leading to its reinforcement with tension-band wiring. On the left side, the lateral bowing and the occlusion of the canal by sclerotic bone after the previous subtrochanteric osteotomy made the insertion of the small Exeter stem impossible. A new rotating and shortening subtrochanteric osteotomy was executed and achieved to restore both the femoral canal alignment and the LLD. Because of the high rate of intraoperative fracture during reaming, and due to the previous osteotomies, the proximal femur was reinforced with cables in advance of its preparation.

## Conclusions

In this case report, the unique undertaking of bilateral, staged THA in a patient suffering from combined short stature, osteochondrodysplasia, and DDH was described. Currently, THA is considered to yield excellent results for the treatment of osteoarthritis secondary to these disorders. However, due to the higher complication rate, the role of meticulous preoperative planning, the right choice of implants, and flawless surgical technique are crucial. The surgeon must be familiar with the variety of pathomorphological alterations and technical THA challenges associated with such cases. The use of cemented femoral stems with minimal offset was an effective method to achieve restoration of hip biomechanics, offering good mid-term clinical results.
